# Supplementary material for: Differentially Represented Proteins in Response to Infection with Mycobacterium tuberculosis Identified by Quantitative Serum Proteomics in Asian Elephants
Source: Pathogens. 2022 Sep 3;11(9):1010. doi: 10.3390/pathogens11091010 (PMC9505326; doi:10.3390/pathogens11091010)
Supplement: Supplementary file 1 [file pathogens-11-01010-s001.zip › Data S2-S5.pdf]

Supplementary Information

Additional file 2. Analysis of immunoglobulin proteins differentially represented in TB+ elephants when compared to TB- animals.

Additional file 3. Gene Ontology (GO) annotations for non-immunoglobulin proteins.

Additional file 4. Most significant pathways identified by Reactome analysis of non-immunoglobulin proteins.

Additional file 5. Protein sequence alignment between *M. tuberculosis* P9WNK5|ESXB_MYCTU ESAT-6-like protein EsxB and identified mycobacterial proteins with predicted antigen binding regions to immunoglobulin proteins.

Additional file 2. Analysis of immunoglobulin proteins differentially represented in TB+ elephants when compared to TB- animals.

Analytical workflow

1. Select immunoglobulin protein sequences differentially represented in TB+ elephants when compared to TB- animals.
2. Paratome (http://www.ofranlab.org) identification of antigen binding regions.
3. Protein BLAST sequence alignment of identified antigen binding regions against *Mycobacterium* (taxid: 1763) using compositional matrix adjustment (https://blast.ncbi.nlm.nih.gov/Blast.cgi?PROGRAM=blastp&PAGE_TYPE=BlastSearch&LINK_LOC=blasthome) to select identifications with highest E-value.

Ig proteins overrepresented in TB+ elephants

G3TYG4
No Blast hits in Paratome. Introduced into the GO annotations and Reactome analysis bellow.

G3UMK9
No Blast hits in Paratome. Introduced into the GO annotations and Reactome analysis bellow.

G3UDI6
paratome_1_tr_G3UDI6_G3UDI6_LOXAF_Ig_like_domain_containing_protein_OS_Loxodonta_africana_OX_9785_PE_4_SV_1

ABR H1: SSISSANYGWS (19-29)

```
RID: 62CNE2PV016
Job Title:H1
Program: BLASTP
Query: H1 ID: lcl|Query_61178(amino acid) Length: 11
Database: nr All non-redundant GenBank CDS
translations+PDB+SwissProt+PIR+PRF excluding environmental samples from WGS
projects

Sequences producing significant alignments:
```

Description Accession	Scientific Name	Common Name	Taxid	Max Score	Total Score	Query cover	E Value	Per. Ident	Acc. Len
DUF3298 domain-containing protein [Mycobacterium sp.] TAM68061.1	Mycobacteriu...	NA	1785	24.4	24.4	63%	52	100.00	264

```
Alignments:

>DUF3298 domain-containing protein [Mycobacterium sp.]
Sequence ID: TAM68061.1 Length: 264
Range 1: 97 to 103

Score:24.4 bits(50), Expect:52,
Method:,
Identities:7/7(100%), Positives:7/7(100%), Gaps:0/7(0%)

Query  3    ISSANYG  9
            ISSANYG
Sbjct  97   ISSANYG  103
```

ABR H2: WLGRGYYSGSSWNRNYGS (41-58)

```
RID: 62D0UFAP016
Job Title:H2
Program: BLASTP
Query: H2 ID: lcl|Query_44192(amino acid) Length: 18
Database: nr All non-redundant GenBank CDS
translations+PDB+SwissProt+PIR+PRF excluding environmental samples from WGS
projects
```

Sequences producing significant alignments:

Description Accession	Scientific Name	Common Name	Taxid	Max Score	Total Score	Query cover	E Value	Per. Ident	Acc. Len
FkbM family methyltransferase [Mycobacterium intracellulare] WP_020822873.1	Mycobacteriu...	NA	1767	27.4	27.4	77%	15	64.29	234

Alignments:

>FkbM family methyltransferase [Mycobacterium intracellulare]
Sequence ID: WP_020822873.1 Length: 234
>hypothetical protein OEM_30260 [Mycobacterium intracellulare subsp. yongonense 05-1390]
Sequence ID: AGP64561.1 Length: 234
>hypothetical protein MOTT12_03025 [Mycobacterium intracellulare subsp. yongonense]
Sequence ID: ARR78689.1 Length: 234
>FkbM family methyltransferase [Mycobacterium intracellulare subsp. chimaera]
Sequence ID: ASL15902.1 Length: 234
>FkbM family methyltransferase [Mycobacterium intracellulare subsp. chimaera]
Sequence ID: PBA56158.1 Length: 234
Range 1: 9 to 20

Score:27.4 bits(57), Expect:15,
Method:,
Identities:9/14(64%), Positives:9/14(64%), Gaps:2/14(14%)

```
Query  1   WLGRGYYSGSSWNR  14
           WL R YYS   WNR
Sbjct  9   WL-R-YYSDRNWNR  20
```

Could not find ABR3

G3UKC4
paratome_1_tr_G3UKC4_G3UKC4_LOXAF_Ig_like_domain_containing_protein_OS_Loxodonta_africana_OX_9785_PE_4_SV_1
Could not find ABR1

ABR H2: WMGGIYPGDSDTCY (66-79)
RID: 62DVU5AF016
Job Title:H2
Program: BLASTP
Query: H2 ID: lcl|Query_96500(amino acid) Length: 14
Database: nr All non-redundant GenBank CDS translations+PDB+SwissProt+PIR+PRF excluding environmental samples from WGS projects

Sequences producing significant alignments:

Description Accession	Scientific Name	Common Name	Taxid	Max Score	Total Score	Query cover	E Value	Per. Ident	Acc. Len
TetR/AcrR family transcriptional regulator [Mycobacterium goodii] MBU8811282.1	Mycobacteriu...	NA	134601	27.8	27.8	71%	5.5	70.00	207
DUF2617 family protein [Mycobacterium asiaticum] WP_065128554.1	Mycobacteriu...	NA	1790	27.8	27.8	64%	5.5	77.78	164

Alignments:

>TetR/AcrR family transcriptional regulator [Mycobacterium goodii]
Sequence ID: MBU8811282.1 Length: 207
>TetR/AcrR family transcriptional regulator [Mycobacterium goodii]
Sequence ID: MBU8817019.1 Length: 207
>TetR/AcrR family transcriptional regulator [Mycobacterium goodii]
Sequence ID: MBU8830908.1 Length: 207

```
Range 1: 194 to 203

Score:27.8 bits(58), Expect:5.5,
Method:,
Identities:7/10(70%), Positives:8/10(80%), Gaps:0/10(0%)

Query  1    WMGGIYPGDS  10
            WMG IY G+S
Sbjct  194  WMGAIYAGES  203
```

>DUF2617 family protein [Mycobacterium asiaticum]
Sequence ID: WP_065128554.1 Length: 164
>hypothetical protein A5645_05960 [Mycobacterium asiaticum]
Sequence ID: OBK97578.1 Length: 164

```
Range 1: 115 to 123

Score:27.8 bits(58), Expect:5.5,
Method:,
Identities:7/9(78%), Positives:9/9(100%), Gaps:0/9(0%)

Query  1    WMGGIYPGD  9
            W+GGI+PGD
Sbjct  115  WLGGIFPGD  123
```

Could not find ABR3

G3TUN8
paratome_1_tr_G3TUN8_G3TUN8_LOXAF_Ig_like_domain_containing_protein_OS_Loxodonta_africana_OX_9785_PE_4_SV_1

ABR H1: PSWVLSQVTLKESGPGLIKPSQTLSLTCTVSGFSLSTSGVGVS (14-56)
No significant similarity found.

ABR H2: WLALIYWDDDKRYSS (68-82)
RID: 62VY1CXE016
Job Title:H2
Program: BLASTP
Query: H2 ID: lcl|Query_82580(amino acid) Length: 15
Database: nr All non-redundant GenBank CDS translations+PDB+SwissProt+PIR+PRF excluding environmental samples from WGS projects

Sequences producing significant alignments:

Description	Scientific Name	Common Name	Taxid	Max Score	Total Score	Query cover	E Value	Per. Ident	Acc. Len	Accession
TetR/AcrR family transcriptional regulator [Mycobacterium simiae]	Mycobacteriu...	NA	1784	27.4	27.4	60%	9.3	66.67	186	WP_149655796.1
PE-PPE domain-containing protein [Mycobacterium palauense]	Mycobacteriu...	NA	2034511	27.4	27.4	66%	9.3	80.00	656	WP_099021260.1
DinB family protein [Mycobacterium tuberculosis]	Mycobacteriu...	NA	1773	26.9	26.9	46%	13	85.71	78	MBZ4317686.1

Alignments:

>TetR/AcrR family transcriptional regulator [Mycobacterium simiae]
Sequence ID: WP_149655796.1 Length: 186
>TetR/AcrR family transcriptional regulator [Mycobacterium simiae]
Sequence ID: KAA1248348.1 Length: 186

```
Range 1: 157 to 168

Score:27.4 bits(57), Expect:9.3,
Method:,
Identities:8/12(67%), Positives:8/12(66%), Gaps:3/12(25%)

Query  2    LALIYW---DDD  10
            L LIYW   DDD
Sbjct  157  LSLIYWPGEDDD  168
```

>PE-PPE domain-containing protein [Mycobacterium palauense]
Sequence ID: WP_099021260.1 Length: 656

```
Range 1: 477 to 485

Score:27.4 bits(57), Expect:9.3,
Method:,
Identities:8/10(80%), Positives:8/10(80%), Gaps:1/10(10%)

Query  1    WLALIYWDDD  10
            WLAL  WDDD
Sbjct  477  WLAL-HWDDD  485
```

>DinB family protein [Mycobacterium tuberculosis]
Sequence ID: MBZ4317686.1 Length: 78
Range 1: 25 to 31

Score:26.9 bits(56), Expect:13,
Method:,
Identities:6/7(86%), Positives:7/7(100%), Gaps:0/7(0%)

```
Query  4    LIYWDDD  10
            LIYW+DD
Sbjct  25   LIYWNDD  31
```

Could not find ABR3

G3TWA7
paratome_1_tr_G3TWA7_G3TWA7_LOXAF_Ig_like_domain_containing_protein_OS_Loxodonta_africana_OX_9785_PE_4_SV_1
Could not find ABR1

ABR L2: LLIYDASNRAS (74-84)
RID: 62VZ5E5M016
Job Title:L2
Program: BLASTP
Query: L2 ID: lcl|Query_46397(amino acid) Length: 11
Database: nr All non-redundant GenBank CDS translations+PDB+SwissProt+PIR+PRF excluding environmental samples from WGS projects

Sequences producing significant alignments:

Description Accession	Scientific Name	Common Name	Taxid	Max Score	Total Score	Query cover	E Value	Per. Ident	Acc. Len
LuxR family transcriptional regulator [Mycobacterium... BBX61765.1	Mycobacteriu...	NA	220927	25.7	25.7	100%	18	60.00	979

Alignments:

>LuxR family transcriptional regulator [Mycobacterium saskatchewanense]
Sequence ID: BBX61765.1 Length: 979
Range 1: 176 to 190

Score:25.7 bits(53), Expect:18,
Method:,
Identities:9/15(60%), Positives:9/15(60%), Gaps:4/15(26%)

```
Query  1    LLIYDASN----RAS  11
            LLIYDA      RAS
Sbjct  176  LLIYDAASTLLERAS  190
```

Could not find ABR3

G3TVW0
paratome_1_tr_G3TVW0_G3TVW0_LOXAF_Ig_like_domain_containing_protein_OS_Loxodonta_africana_OX_9785_PE_4_SV_1
Could not find ABR1

ABR L2: LLIYDASSRAS (75-85)
RID: 62W6CRYA013
Job Title:L2
Program: BLASTP
Query: L2 ID: lcl|Query_74216(amino acid) Length: 11
Database: nr All non-redundant GenBank CDS translations+PDB+SwissProt+PIR+PRF excluding environmental samples from WGS projects

Sequences producing significant alignments:

Description Accession	Scientific Name	Common Name	Taxid	Max Score	Total Score	Query cover	E Value	Per. Ident	Acc. Len
LuxR family transcriptional regulator [Mycobacterium... BBX61765.1	Mycobacteriu...	NA	220927	28.2	28.2	100%	2.2	66.67	979

Alignments:

>LuxR family transcriptional regulator [Mycobacterium saskatchewanense]
Sequence ID: BBX61765.1 Length: 979
Range 1: 176 to 190

Score:28.2 bits(59), Expect:2.2,
Method:,
Identities:10/15(67%), Positives:10/15(66%), Gaps:4/15(26%)

```
Query  1    LLIYDASS----RAS  11
            LLIYDA S    RAS
Sbjct  176  LLIYDAASTLLERAS  190
```

Could not find ABR3

G3TT51
paratome_1_tr_G3TT51_G3TT51_LOXAF_Ig_like_domain_containing_protein_OS_Loxodonta_africana_OX_9785_PE_4_SV_1
Could not find ABR1

ABR L2: LLIYGASSRASR (70-81)
RID: 62W75XZ301R
Job Title:L2
Program: BLASTP
Query: L2 ID: lcl|Query_193298(amino acid) Length: 12
Database: nr All non-redundant GenBank CDS translations+PDB+SwissProt+PIR+PRF excluding environmental samples from WGS projects

Sequences producing significant alignments:

Description Accession	Scientific Name	Common Name	Taxid	Max Score	Total Score	Query cover	E Value	Per. Ident	Acc. Len
LuxR family transcriptional regulator [Mycobacterium... BBX61765.1	Mycobacteriu...	NA	220927	26.9	44.1	100%	7.6	62.50	979

Alignments:

>LuxR family transcriptional regulator [Mycobacterium saskatchewanense]
Sequence ID: BBX61765.1 Length: 979
Range 1: 176 to 191

Score:26.9 bits(56), Expect:7.6,
Method:,
Identities:10/16(63%), Positives:10/16(62%), Gaps:4/16(25%)

```
Query  1    LLIYGASS----RASR  12
            LLIY A S    RASR
Sbjct  176  LLIYDAASTLLERASR  191
```

Could not find ABR3

G3TVJ6
paratome_1_tr_G3TVJ6_G3TVJ6_LOXAF_Ig_like_domain_containing_protein_OS_Loxodonta_africana_OX_9785_PE_4_SV_1
Could not find ABR1

ABR L2: LLIYEASNRAS (72-82)
RID: 62WC16FZ013
Job Title:L2
Program: BLASTP
Query: L2 ID: lcl|Query_11729(amino acid) Length: 11
Database: nr All non-redundant GenBank CDS translations+PDB+SwissProt+PIR+PRF excluding environmental samples from WGS projects

Sequences producing significant alignments:

Description Accession	Scientific Name	Common Name	Taxid	Max Score	Total Score	Query cover	E Value	Per. Ident	Acc. Len
hypothetical protein [Mycobacterium colombiense] WP_076051897.1	Mycobacteriu...	NA	339268	25.7	25.7	100%	18	72.73	390

Alignments:

>hypothetical protein [Mycobacterium colombiense]
Sequence ID: WP_076051897.1 Length: 390
>hypothetical protein A5739_08795 [Mycobacterium colombiense]
Sequence ID: OMC33428.1 Length: 390

Range 1: 135 to 145

Score:25.7 bits(53), Expect:18,
Method:,
Identities:8/11(73%), Positives:9/11(81%), Gaps:0/11(0%)

```
Query  1    LLIYEASNRAS  11
            L I+EA NRAS
Sbjct  135  LWIFEATNRAS  145
```

ABR L3: FQNYY (115-119)

RID: 62WD1HTK013
Job Title:L3
Program: BLASTP
Query: L3 ID: lcl|Query_20252(amino acid) Length: 5
Database: nr All non-redundant GenBank CDS translations+PDB+SwissProt+PIR+PRF excluding environmental samples from WGS projects

Sequences producing significant alignments:

Description Accession	Scientific Name	Common Name	Taxid	Max Score	Total Score	Query cover	E Value	Per. Ident	Acc. Len
type I site-specific deoxyribonuclease, HsdR family... SGD45129.1	Mycobacteriu...	NA	1773	22.3	22.3	100%	73	100.00	1040
type I restriction endonuclease subunit R [Mycobacterium... WP_065474021.1	Mycobacteriu...	NA	1780	22.3	22.3	100%	73	100.00	1033

Alignments:

>type I site-specific deoxyribonuclease, HsdR family [Mycobacterium tuberculosis]
Sequence ID: SGD45129.1 Length: 1040

Range 1: 636 to 640

Score:22.3 bits(45), Expect:73,
Method:,
Identities:5/5(100%), Positives:5/5(100%), Gaps:0/5(0%)

```
Query  1    FQNYY  5
            FQNYY
Sbjct  636  FQNYY  640
```

>type I restriction endonuclease subunit R [Mycobacterium malmoense]
Sequence ID: WP_065474021.1 Length: 1033
>DEAD/DEAH box helicase [Mycobacterium malmoense]
Sequence ID: OCB21517.1 Length: 1033

Range 1: 677 to 681

Score:22.3 bits(45), Expect:73,
Method:,
Identities:5/5(100%), Positives:5/5(100%), Gaps:0/5(0%)

```
Query  1    FQNYY  5
            FQNYY
Sbjct  677  FQNYY  681
```

Ig proteins underrepresented in TB+ elephants

G3UIN4

paratome_1_tr_G3UIN4_G3UIN4_LOXAF_Ig_like_domain_containing_protein_OS_Loxodonta_africana_OX_9785_PE_4_SV_1

ABR L1: QSLFDSSDKKDYLN (27-40)

RID: 62WE6VB3016
Job Title:L1
Program: BLASTP
Query: L1 ID: lcl|Query_11081(amino acid) Length: 14
Database: nr All non-redundant GenBank CDS translations+PDB+SwissProt+PIR+PRF excluding environmental samples from WGS projects

Sequences producing significant alignments:

Description Accession	Scientific Name	Common Name	Taxid	Max Score	Total Score	Query cover	E Value	Per. Ident	Acc. Len
RND family transporter [Mycobacterium sp. E2479] WP_067937080.1	Mycobacteriu...	NA	1834134	26.5	45.8	92%	16	72.73	954
restriction endonuclease [Mycobacterium sp. KBS0706] TSD87553.1	Mycobacteriu...	NA	2578109	26.5	44.1	78%	16	75.00	1376

Alignments:

> Resistance-nodulation-division (RND) family transporter [Mycobacterium sp. E2479]
Sequence ID: WP_067937080.1 Length: 954
Membrane protein, transporter, associated to drug resistance
>hypothetical protein A5686_08675 [Mycobacterium sp. E2479]
Sequence ID: OBH53630.1 Length: 954

```
Range 1: 590 to 600

Score:26.5 bits(55), Expect:16,
Method:,
Identities:8/11(73%), Positives:8/11(72%), Gaps:0/11(0%)

Query  1    QSLFDSSDKKD  11
            QSLFD  DK D
Sbjct  590  QSLFDATDKID  600

Range 2: 669 to 680

Score:19.3 bits(38), Expect:6160,
Method:,
Identities:8/13(62%), Positives:8/13(61%), Gaps:4/13(30%)

Query  4    FDSSDKKD---YL  13
            FDSS K D   YL
Sbjct  669  FDSS-KNDEFFYL  680
```

>restriction endonuclease/methylase [Mycobacterium sp. KBS0706]
Sequence ID: TSD87553.1 Length: 1376

Range 1: 867 to 878

Methylation, anti-mycobacterial activity in response to vaccination correlates with altered DNA methylation

```
Score:26.5 bits(55), Expect:16,
Method:,
Identities:9/12(75%), Positives:9/12(75%), Gaps:2/12(16%)

Query  3    LFDSSD--KKDY  12
            LFDSSD   KDY
Sbjct  867  LFDSSDPLTKDY  878

Range 2: 737 to 742

Score:17.6 bits(34), Expect:25377,
Method:,
Identities:5/6(83%), Positives:5/6(83%), Gaps:0/6(0%)

Query  2    SLFDSS  7
            SLFD S
Sbjct  737  SLFDGS  742
```

ABR L2: LLIYLASKRDS (52-62)

RID: 62WS463J013
Job Title:L2
Program: BLASTP
Query: L2 ID: lcl|Query_21455(amino acid) Length: 11

Database: nr All non-redundant GenBank CDS translations+PDB+SwissProt+PIR+PRF excluding environmental samples from WGS projects

Sequences producing significant alignments:

Description	Scientific Name	Common Name	Taxid	Max Score	Total Score	Query cover	E Value	Per. Ident	Acc. Len	Accession
protein translocase subunit SecD [Mycobacterium sp.]	Mycobacteriu...	NA	1785	24.8	24.8	63%	36	100.00	609	MBV9350256.1

Alignments:

>protein translocase subunit SecD [Mycobacterium sp.] Sequence ID: MBV9350256.1 Length: 609

Range 1: 563 to 569

Transmembrane transport, cell inner membrane, secretion of mycobacterial proteins linked to pathogenicity

Score:24.8 bits(51), Expect:36,
Method:,
Identities:7/7(100%), Positives:7/7(100%), Gaps:0/7(0%)

```
Query  2    LIYLASK  8
            LIYLASK
Sbjct  563  LIYLASK  569
```

Could not find ABR3

Additional file 3. Gene Ontology (GO) annotations for non-immunoglobulin proteins.

Analytical workflow

1. Select non-immunoglobulin protein sequences differentially represented in TB+ elephants when compared to TB- animals.
2. Uniprot (https://www.uniprot.org) GO annotations for Molecular function, Biological process, and Subcellular localization in human homolog proteins.

**Proteins overrepresented in TB+ elephants**		
**Proteins identified**	**Human homolog**	**GO annotations:**<br>**Molecular function (MF)**<br>**Biological process (BP)**<br>**Subcellular localization (CC)**
Christmas factor (F9)	P00740	MF: Calcium ion binding, endopeptidase<br>BP: Blood coagulation, proteolysis<br>CC: Extracellular region or secreted
Glycoprotein V platelet (GP5)	P40197	MF: Platelet adhesion to blood vessels<br>BP: Blood coagulation, cell adhesion, positive regulation of platelet activation<br>CC: Membrane
Apolipoprotein C-III (APOC3)	P02656	MF: Cholesterol binding, lipase inhibitor, lipoprotein binding<br>BP: Negative regulation of lipid metabolism and low-density lipoprotein particle clearance<br>CC: Secreted
Apolipoprotein B (APOB)	P04114	MF: Cholesterol transfer, heparin/lipase/phospholipid binding<br>BP: Artery morphogenesis, fertilization, positive regulation of cholesterol metabolism and gene expression, response to virus<br>CC: Extracellular region or secreted, cytoplasm
Apolipoprotein D (APOD)	P05090	MF: Cholesterol binding, lipid transport<br>BP: Aging, glucose and lipid metabolism, negative regulation of cytokines involved in inflammatory response and T cell migration, response to axon injury and reactive oxygen species<br>CC: Extracellular region or secreted
Joining chain of multimeric IgA and IgM (JCHAIN)	P01591	MF: Antigen binding, IgA binding<br>BP: Adaptive and humoral immune response, antibacterial humoral response<br>CC: Secreted
Immunoglobulin heavy constant mu (IGHM)	P01871	MF: Antigen binding<br>BP: Adaptive and innate immune response, antibacterial humoral response<br>CC: Extracellular region or secreted, plasma membrane

Biotinidase (BTD)	P43251	MF: Biotinidase activity<br>BP: Biotin metabolism, central nervous system development<br>CC: Extracellular region or secreted
Tenascin C (TNC)	P24821	MF: Extracellular matrix structural constituent<br>BP: Cell adhesion, positive regulation of gene expression, regulation of cell growth and inflammatory response, response to wounding<br>CC: Extracellular region or secreted
CD5 molecule like (CD5L)	O43866	MF: Scavenger receptor activity, endopeptidase<br>BF: Immune cellular defense response, apoptosis, inflammatory response<br>CC: Extracellular region or secreted, cytoplasm
Glycosyl-phosphatidylinositol-specific phospholipase D (GPLD1)	P80108	MF: Sodium channel regulator activity<br>BP: Positive regulation of apoptosis, cellular response to pH, cholesterol, calcium and insulin<br>CC: Extracellular region or secreted
Complement C5 (C5)	P01031	MF: Chemokine, endopeptidase inhibitor and signaling receptor activity<br>BP: Complement activation, inflammatory response, positive regulation of angiogenesis and chemokine production<br>CC: Extracellular region or secreted
**Proteins underrepresented in TB+ elephants (overrepresented in TB- elephants)**		
Alpha-1-B glycoprotein (A1BG)	P04217	MF: Platelet and neutrophil degranulation<br>BP: Leukocyte immunoglobulin-like receptor<br>CC: Extracellular region or secreted
Serpin family A1 (SERPINA1) Encoding Alpha-1 antitrypsin (AAT)	P01009	MF: Serin-type endopeptidase inhibitor activity<br>BP: Acute-phase response, blood coagulation, negative regulation of endopeptidase activity<br>CC: Extracellular region or secreted, endoplasmic reticulum
Transthyretin (TTR)	P02766	MF: Hormone activity, thyroid hormone binding<br>BP: Purine nucleobase and retinol metabolic processes, thyroid hormone transport<br>CC: Extracellular region or secreted, cytoplasm
Keratin 10 (KRT10)	P60014	MF: Identical protein binding<br>BP: Formation of rigid and resistant hair<br>CC: Cytoskeleton

Additional file 4. Most significant pathways identified by Reactome analysis of non-immunoglobulin proteins.

Analytical workflow

1. Select non-immunoglobulin protein sequences differentially represented in TB+ elephants when compared to TB- animals.
2. Reactome (https://reactome.org/PathwayBrowser/) identification and characterization of biological pathways. As disclosed in the webpage, Reactome is a curated database of pathways and reactions in human biology. Reactions are considered as pathway 'steps'. Reactome defines a 'reaction' as any event in biology that changes the state of a biological molecule. Binding, activation, translocation, degradation and classical biochemical events involving a catalyst are all reactions. Reactome's annotated data describe reactions possible if all annotated proteins and small molecules were present and active simultaneously in a cell. By overlaying an experimental dataset on these annotations, a pathway over-representation analysis can be done. A binomial test is used to calculate the probability shown for each result, and the p-values are corrected for the multiple testing (Benjamini–Hochberg procedure) that arises from evaluating the submitted list of identifiers against every pathway. This is an overrepresentation analysis: A statistical (hypergeometric distribution) test that determines whether certain Reactome pathways are over-represented (enriched) in the submitted data. It answers the question 'Does my list contain more proteins for pathway X than would be expected by chance?' This test produces a probability score, which is corrected for false discovery rate using the Benjamani-Hochberg method.

**Proteins overrepresented in TB+ elephants**					
**Pathways**	**Found Ratio**	**p<0.05**	**FDR**	**Reactions Found Ratio**	**Proteins identified**
Defective F9 activation	2/7<br>4.6e-04	1.4e-05	0.001	1/1<br>7.2e-05	Christmas factor (F9)<br>Glycoprotein V platelet (GP5)
Defective F9 causes hemophilia B	2/13<br>8.6e-04	4.9e-05	0.002	4/4<br>2.9e-04	F9, GP5
Chylomicron assembly	2/14<br>2.3e-04	5.6e-05	0.002	5/5<br>3.6e-04	Apolipoprotein C-III (APOC3)<br>Apolipoprotein B (APOB)
Chylomicron remodeling	2/21<br>0.001	8.3e-05	0.002	3/3<br>2.2e-04	APOC3, APOB
Defects of contact activation system (CAS) and kallikrein/kinin system (KKS)	2/21<br>0.001	1.3e-04	0.002	6/18<br>0.001	F9, GP5
Diseases of hemostasis	2/21<br>0.001	1.3e-04	0.002	6/18<br>0.001	F9, GP5
Intrinsic pathway of fibrin clot formation	2/26<br>0.002	1.9e-04	0.003	8/24<br>0.002	F9, GP5
Hemostasis	5/803<br>0.053	2.4e-04	0.003	23/334<br>0.024	F9, GP5, APOB<br>Joining chain of multimeric IgA and IgM (JCHAIN)

					Immunoglobulin heavy constant mu (IGHM)
Plasma lipoproteins assembly	2/30<br>0.002	2.6e-04	0.003	10/19<br>0.001	APOC3, APOB
Formation of fibrin clot (clotting cascade)	2/43<br>0.003	5.2e-04	0.005	9/61<br>0.004	F9, GP5
Defective F9 secretion	1/1<br>6.6e-05	7.9e-04	0.007	1/1<br>7.2e-05	F9
Plasma lipoprotein remodeling	2/56<br>0.004	8.8e-04	0.007	6/31<br>0.002	APOC3, APOB
Cell surface interactions at the vascular wall	3/257<br>0.017	9.7e-04	0.007	3/65<br>0.005	APOB, JCHAIN
NR1H3 & NR1H2 regulate gene expression linked to cholesterol transport and efflux	2/66<br>0.004	0.001	0.009	2/40<br>0.003	Apolipoprotein D (APOD)
Defective BTD causes biotidinase deficiency	1/2<br>1.3e-04	0.002	0.009	2/2<br>1.4e-04	Biotinidase (BTD)
Retinoid metabolism transport	2/79<br>0.005	0.002	0.009	9/28<br>0.002	APOC3, APOB
NR1H2 and NR1H3-mediated signaling	2/85<br>0.006	0.002	0.01	2/60<br>0.004	APOD
Metabolism of fat-soluble vitamins	2/89<br>0.006	0.002	0.011	9/34<br>0.002	APOC3, APOB
Plasma lipoprotein assembly, remodeling, and clearance	2/102<br>0.007	0.003	0.012	30/85<br>0.006	APC3, APOB
Metabolism of vitamins and cofactors	3/382<br>0.025	0.003	0.012	11/204<br>0.015	APOC3, APOB
Defective cofactor function of FVIIIa variant	1/4<br>2.6e-04	0.003	0.012	1/1<br>7.2e-05	APOC3, APOB, BTD
Defective F9 causes thrombophilia	1/4<br>2.6e-04	0.003	0.012	1/1<br>7.2e-05	F9
Defective F9 variant does not activate FX	1/4<br>2.6e-04	0.003	0.012	1/1<br>7.2e-05	F9
Post-translational protein phosphorylation	2/109<br>0.007	0.003	0.012	1/1<br>7.2e-05	Tenascin C (TNC)<br>APOB
Defective gamma-carboxylation of F9	1/5<br>3.3e-04	0.004	0.012	1/1<br>7.2e-05	F9
**Proteins underrepresented in TB+ elephants**					
**Pathway**	**Found Ratio**	**p-value**	**FDR***	**Reactions Found Ratio**	**Proteins identified**
Neutrophil degranulation	3/480<br>0.041	0.001	0.019	3/10<br>7.2e-04	Alpha-1-B glycoprotein (A1BG)<br>Serpin family A1 (SERPINA1)<br>Transthyretin (TTR)
Platelet degranulation	2/128<br>0.011	0.002	0.019	1/11<br>8.0e-04	A1BG, SERPINA1
Formation of the cornified envelope	2/129<br>0.011	0.002	0.019	8/27<br>0.002	Keratin 10 (KRT10)
Response to elevated platelet cytosolic Ca2+	2/133<br>0.011	0.002	0.019	1/14<br>0.001	A1BG, SERPINA1

Hemostasis	3/726 0.063	0.004	0.027	7/334 0.024	A1BG, SERPINA1
Keratinization	2/217 0.019	0.005	0.027	15/34 0.002	KRT10
Retinoid cycle disease events	1/13 0.001	0.007	0.027	1/16 0.001	TTR
Diseases of the neuronal system	1/13 0.001	0.007	0.027	1/17 0.001	TTR
Diseases associated with visual transduction	1/13 0.001	0.007	0.027	1/17 0.001	TTR
Platelet activation, signaling and aggregation	2/265 0.023	0.007	0.029	1/116 0.008	A1BG, SERPINA1
Common pathway of fibrin clot formation	1/22 0.002	0.011	0.034	4/29 0.002	SERPINA1
The canonical retinoid cycle in rods (twilight vision)	1/23 0.002	0.012	0.034	2/25 0.002	TTR
Intrinsic pathway of fibrin clot formation	1/23 0.002	0.012	0.034	2/24 0.002	SERPINA1
Cargo concentration in the ER	1/33 0.003	0.017	0.034	2/12 8.7e-04	SERPINA1
Innate immune system	3/1195 0.103	0.017	0.034	3/170 0.051	A1BG, SERPINA1, TTR
Formation of fibrin clot (clotting cascade)	1/39 0.003	0.02	0.035	6/61 0.004	SERPINA1
Retinoid metabolism and transport	1/44 0.004	0.023	0.035	1/28 0.002	TTR
Metabolism of fat-soluble vitamins	1/48 0.004	0.025	0.035	1/34 0.002	TTR
Non-integrin membrane-ECM interactions	1/59 0.005	0.03	0.035	1/22 0.002	TTR
COPII-mediated vesicle transport	1/68 0.006	0.035	0.035	10/16 0.001	SERPINA1
Amyloid fiber formation	1/81 0.007	0.041	0.041	2/33 0.002	TTR

*FDR, False discovery rate.

Additional file 5. Protein sequence alignment between *M. tuberculosis* P9WNK5|ESXB_MYCTU ESAT-6-like protein EsxB and identified mycobacterial proteins with predicted antigen binding regions to immunoglobulin proteins.

Analytical workflow

1. Select immunoglobulin protein sequences differentially represented in TB+ elephants when compared to TB- animals.
2. COBALT (https://www.ncbi.nlm.nih.gov/tools/cobalt/cobalt.cgi?CMD=Web) protein sequence alignment between identified mycobacterial proteins with predicted antigen binding regions to immunoglobulin proteins and *M. tuberculosis* P9WNK5|ESXB_MYCTU ESAT-6-like protein EsxB identified as highly reactive to antibodies in infected elephants (Lyashchenko et al., 2018).

```
>sp|P9WNK5|ESXB_MYCTU ESAT-6-like protein EsxB OS=Mycobacterium
tuberculosis (strain ATCC 25618 / H37Rv) OX=83332 GN=esxB PE=1 SV=1
MAEMKTDAATLAQEAGNFERISGDLKTQIDQVESTAGSLQGQWRGAAGTAAQAAVVRFQEAANKQKQELDEISTN
IRQAGVQYSRADEEQQQALSSQMGF
```

Ig proteins overrepresented in TB+ elephants

NCBI Multiple Sequence Alignment Viewer, Version 1.22.0

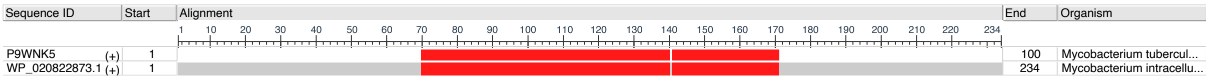


```
P9WNK5.1          1    ---------------------------------------------------------------------MAEMKTDAATL  11
WP_020822873.1    1    MARSLPSGWLRYYSDRNWNRQIVDQLKSHQVNLVLDVGANAGQYGAGLRRATYKDRIVSFEPVSQPFSVLEHEAAADPLW  80

P9WNK5.1          12   AQEAGNFERISGDLKTQIDQVESTAGSLQGQWRGAAGTAAQAAVVRFQEAANKQKQELD-EISTNIRQAGVQYSRADEEQ  90
WP_020822873.1    81   ECRRHALGNIDGEISINVAGNAGQSSSVLGMLKSHQDAFPPANYIGTEEVPMHRLDSVAAEIFRPTDVTFLKIDVQGFEK  160

P9WNK5.1          91   QQALSSQMGF----------------------------------------------------------------  100
WP_020822873.1    161  EVLTGGESTVRDRCVGMQIELSFLHLYEGDMLIHEALDLLHSMGFTLTGLLPGFADPRNGRMLQADGIFFREDD  234
```

NCBI Multiple Sequence Alignment Viewer, Version 1.22.0

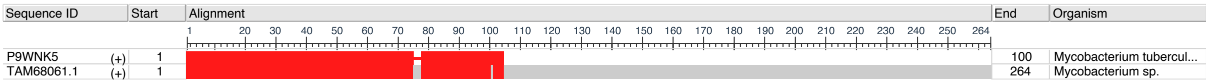


```
P9WNK5.1      1    MAEMKTDAATLAQEAGNFERISGDLKTQIDQVESTAGSLQGQWRGAAGTAAQAAVVRFQEAANKQKQELDEIST---NIR  77
TAM68061.1    1    MRSFTVAASATALVLFGSAAIAAAAPPKDYCAELKGANTGQACRIQMADAGYNVDISFPVSYPDEKSVADFITKERDDFL  80

P9WNK5.1      78   QAGVQYSRADEEQQQALSS-QMGF--------------------------------------------------------  100
TAM68061.1    81   NVAKSSAPRDQPYQLTISSANYGSAIPPRGTEAVVLKVVENVGAHPQTTFKSFNWDQTYRKAIVWTAAADDKNNTPLWRV  160

P9WNK5.1           --------------------------------------------------------------------------------
TAM68061.1    161  DDPLQTVAPIVQSELQKQTAPPATPTPPAPPANQTQPPPPASPTQTPPPVTIAPAAAYDPANYQNFAVTNDGVIFFFDQG  240

P9WNK5.1           ------------------------
TAM68061.1    241  RMLPDAAGATQVLVPRSAIDPMLA  264
```

NCBI Multiple Sequence Alignment Viewer, Version 1.22.0

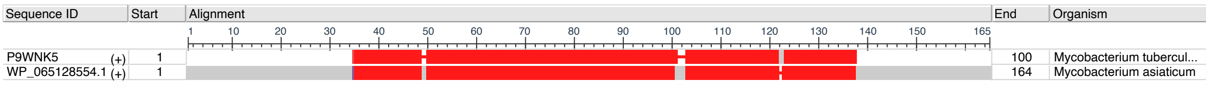


```
P9WNK5.1          1    ----------------------------------MAEMKTDAATLAQE-AGNFERISGDLKTQIDQVESTAGSLQGQWRG  45
WP_065128554.1    1    MPLHQLAVAPADVSGARLALALNTVAPDPLATCRLAHPGGGALLLGVLGASHVVTVEHGTTRFSEQVSCTAGDELPSRSG  80

P9WNK5.1          46   AAGTAAQAAVVRFQEAANKQ--KQELDEISTNIRQAGVQYSRADEEQQQALSSQMGF----------------------  100
WP_065128554.1    81   APGYWIEARTEKHGEASFRALASRLRDRCAKEVGWLGGIFP-GDDAALTALAAEPDGNGWQWQTWHLYPSSAGGTVVHTA  159

P9WNK5.1               -----
WP_065128554.1    160  SRWHP  164
```

NCBI Multiple Sequence Alignment Viewer, Version 1.22.0

Sequence ID	Start	Alignment	End	Organism
P9WNK5 (+)	1		100	Mycobacterium tubercul...
OBK97578.1 (+)	1		164	Mycobacterium asiaticum

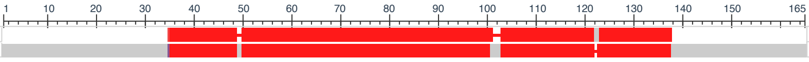


```
P9WNK5.1     1    ----------------------------------MAEMKTDAATLAQE-AGNFERISGDLKTQIDQVESTAGSLQGQWRG  45
OBK97578.1   1    MPLHQLAVAPADVSGARLALALNTVAPDPLATCRLAHPGGGALLLGVLGASHVVTVEHGTTRFSEQVSCTAGDELPSRSG  80

P9WNK5.1     46   AAGTAAQAAVVRFQEAANKQ--KQELDEISTNIRQAGVQYSRADEEQQQALSSQMGF-----------------------  100
OBK97578.1   81   APGYWIEARTEKHGEASFRALASRLRDRCAKEVGWLGGIFP-GDDAALTALAAEPDGNGWQWQTWHLYPSSAGGTVVHTA  159

P9WNK5.1          -----
OBK97578.1   160  SRWHP  164
```

NCBI Multiple Sequence Alignment Viewer, Version 1.22.0

Sequence ID	Start	Alignment	End	Organism
P9WNK5 (+)	1		100	Mycobacterium tubercul...
WP_076051897.1 (+)	1		390	Mycobacterium colombi...

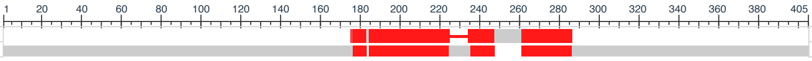


```
P9WNK5.1             --------------------------------------------------------------------------------
WP_076051897.1  1    MSRIFDDFDRTDSSPAGDTESSYHFLNRVARPEWALVRDLVDEWFSEYPVAAQPDLRSRLRDDDYVQHIGAWWELYIYRL  80

P9WNK5.1             --------------------------------------------------------------------------------
WP_076051897.1  81   FRRLGYDVSIHPVLADTSRQPDFLATREDSSIYVECVVYVSNTGAVHGEGSGERLWIFEATNRASDPNFLLDIEIHQAAA  160

P9WNK5.1        1    ---------------MAEMKTDAATLAQEAGNFERISGDLKTQIDQVESTAGSLQGQWRGAAGT----------AAQAAV  55
WP_076051897.1  161  QRPKASEIIRPLENWLSSLDPD-EVLEQIAAGAGAPEFVLEVRGWTIEYSAWPVKPESRGEGGRLIGIYPMIGGSINGEM  239

P9WNK5.1        56   VRFQEAANKQKQELDEISTNIRQAGVQYSRADEEQQQALSSQMGF-----------------------------------  100
WP_076051897.1  240  LRFQEI--------------VRRKGGRYGLPDKPFVVAVLNTSAFLDEGDLAEALFGSVVIEYYEGVADSVRRVRQRDGY  305

P9WNK5.1             --------------------------------------------------------------------------------
WP_076051897.1  306  WRQGPPKRGSRVSGVLVGKDIYPWTVSRSELPELWVNPWSDIPMDHHLPFTVRTANDEGHVFRIRQDGTSTDMLFGLTPG  385

P9WNK5.1             -----
WP_076051897.1  386  WPGFD  390
```

NCBI Multiple Sequence Alignment Viewer, Version 1.22.0

Sequence ID	Start	Alignment	End	Organism
P9WNK5 (+)	1		100	Mycobacterium tubercul...
SGD45129.1 (+)	1		1,040	Mycobacterium tubercul...

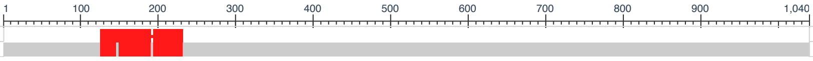


```
P9WNK5.1          --------------------------------------------------------------------------------
SGD45129.1   1    MTQQTKTIAESNNFIVLDKYTARQDGGDRYQSEEELERELIQDLRNQGYEYLPDLTSPSAMLANVRTQLQVLNNVVFTDN  80

P9WNK5.1     1    -------------------------------------------MAEMKTDAATLAQEAGNFER---ISGDLKTQIDQVES  34
SGD45129.1   81   EWRRFTEEYLDNPGDTIIDKTRKIHTDYICDFTFDDGRLDNIYLADKKKPVRNKVQVIRQFEQTGTHANRYDVTILVNGL  160

P9WNK5.1     35   TAGSLQGQWRGAAGTAAQAAVVRFQEAA----NKQKQELDEISTNIRQAGVQYSRADEEQQQALSSQMGF----------  100
SGD45129.1   161  PLVQIELKKRGVAIREAFNQIHRYSKESFNTDNSLFKYLQVFVISNGTDTRYFANTTKRDKNSFDFTMNWARSDNTLIKD  240
```

NCBI Multiple Sequence Alignment Viewer, Version 1.22.0

Sequence ID	Start	Alignment	End	Organism
P9WNK5 (+)	1		100	Mycobacterium tubercul...
OCB21517.1 (+)	1		1,033	Mycobacterium malmoe...

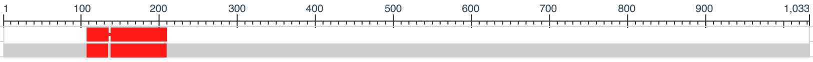


```
P9WNK5.1          --------------------------------------------------------------------------------
OCB21517.1   1    MTEAKAVRYDLIAVSAESTVVAKFVADAKTATEYQSEAQLEREFIKILQSQEYEYLKITTELDLIANLRSQLEALNAITF  80

P9WNK5.1     1    ------------------------MAEMKTDAATLAQEAGNFERISGDLKTQI--DQVESTAGSLQGQWRGAAGTAAQAA  54
OCB21517.1   81   SDSEWKRFFTEKIAGANEGIEDKTVRVQEDYVQLLTRDDGSIKNVKLIDKTNIHDNKLQVINQYEVGQSEGGAKYANRYD  160

P9WNK5.1     55   VVRFQEAANKQKQELDEISTNIRQAGVQYSRADEEQQQALSSQMGF----------------------------------  100
OCB21517.1   161  VTVLVNGLPMVHIELKRRGVDIREAFNQINRYQRDSFWAGSGLFDYVQLFVISNGTLTKYYSNTTRRQHISEAAGSKKAK  240
```

Ig protein underrepresented in TB+ elephants

NCBI Multiple Sequence Alignment Viewer, Version 1.22.0

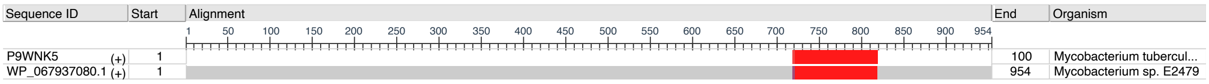


```
P9WNK5.1         1    -----------------------------------------------------------------------------MAE  3
WP_067937080.1   641  ILTEHNTIVPQLAQLDELSRQMMDLGYAFDSSKNDEFFYLPPDAFSNPYFQIDLRFFVSPDGKAARYMVYHDGEALTPEG  720

P9WNK5.1         4    MKTDAATLAQEAGNFERIS-GDLKTQIDQVESTAGSLQGQWRGAAGTAAQAAVVRFQEAANKQKQELDEISTNIRQAGVQ  82
WP_067937080.1   721  IKHDQTYLPAAKEALKGTALAGARVYLGGAAATYWDIQEAARMDLIIAATAAFALIFLVMLLITRSVIAALVIVGTVAFS  800

P9WNK5.1         83   YSRADEEQQQALSSQMGF--------------------------------------------------------------  100
WP_067937080.1   801  YSGAFGLSVLVWQHFLGIPLSWLNLPLTFIILVAVGSDYNLLLIVRYLEESKAGLNTGLIRAVANSGKVVTTAGLVFAVT  880
```

NCBI Multiple Sequence Alignment Viewer, Version 1.22.0

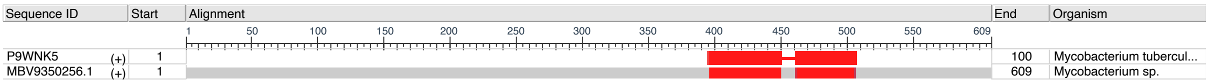


```
P9WNK5.1       1    --------------------------------------------------------------------------MAEMKT  6
MBV9350256.1   321  KSAAADTWADFTAAHVGTQTAFTLDSRVVSAPQIREAIPGGRTQITGGDPPFTADTARQLANVLKYGSLPLSFESSEAET  400

P9WNK5.1       7    DAATLAQEAGNFERISGDLKTQIDQVESTAGSLQGQWRGAAGTAAQAAV-----------VRFQEAANKQKQELDEISTN  75
MBV9350256.1   401  VSATLGLTSLRAGLIAGAIGLALVLLYSLAYYRVLGLLTALSLVASGAMVFAILVLLGRYINYTLDLAGIAGLIIGIGTT  480

P9WNK5.1       76   IRQAGVQYSRADEEQQQALSSQMGF-------------------------------------------------------  100
MBV9350256.1   481  ADSFVVFFERIKDEIREGRSFRSAVPRGWARARKTIVSGNAVTFLAAAVLYFLAIGQVKGFAFTLGLTTILDLVVVFLVT  560
```
